# Predicting Therapeutic Response in Pediatric Ulcerative Colitis—A Journey Towards Precision Medicine

**DOI:** 10.3389/fped.2021.634739

**Published:** 2021-02-17

**Authors:** Ruben J. Colman, Jasbir Dhaliwal, Michael J. Rosen

**Affiliations:** ^1^Division of Gastroenterology, Hepatology and Nutrition, Cincinnati Children's Hospital Medical Center, Cincinnati, OH, United States; ^2^Department of Pediatrics, University of Cincinnati College of Medicine, Cincinnati, OH, United States

**Keywords:** personalized medicine, artificial intelligence, prognosis, protect, companion diagnostic, biomarker, inflammatory bowel disease

## Abstract

Ulcerative colitis (UC) is a disabling disease, characterized by chronic inflammation of the colon, with a rising prevalence worldwide in the pediatric age group. Although UC presents in children with varying severity, disease extent, and comorbidities, initial treatment is essentially uniform, consisting of 5-aminosalicylate drugs with corticosteroid induction for those with moderately to severely active disease. With the advent of anti-tumor necrosis factor (TNF) biologic therapy and several new biologics and small-molecule drugs for UC, precision medicine approaches to treatment are needed to more rapidly achieve sustained remission, restore quality of life, normalize development, and limit exposure to toxic corticosteroids in children with UC. Here, we review available data on clinical, biochemical, histopathologic, and molecular predictors of treatment response in UC. We also address known predictors and special treatment considerations in specific relevant scenarios such as very-early-onset UC, acute severe UC, ileal pouch anal anastomosis, and UC with concomitant primary sclerosing cholangitis. The review concludes with a prediction of how machine learning will integrate multimodal patient data to bring precision medicine to the bedside of children with UC in the future.

## Introduction

Ulcerative colitis (UC) is one of the two distinct entities of chronic inflammatory bowel disease (IBD), for which the exact pathogenesis remains to be elucidated. Increasing understanding of the underlying biologic pathways and recent innovations have led to many new available treatment options, although few have been studied extensively in pediatric patients. With a rapidly growing therapeutic armamentarium, it is becoming critical that we learn to recognize the clinical, histopathologic, molecular, and microbial heterogeneity of UC, so that precision medicine treatment strategies can be tailored to each patient to achieve and maintain optimal outcomes.

## Pediatric UC Epidemiology and Disease Features

Worldwide, the prevalence of UC is rising, and while the incidence is stabilizing in much of the Western world, rates are increasing in newly industrialized countries, specifically in parts of South Asia (1–5). To address the increasing disease burden, there is an urgent need to develop biomarkers that predict disease severity and disease course in a wide diverse population (2).

Features indicative of pediatric UC are well-described in the criteria set forth by the North American and European Societies for Pediatric Gastroenterology Hepatology and Nutrition (NASPGHAN and ESPGHAN, respectively) (6–8). UC typically presents with a continuous inflammation of the rectum and colon proximally and is further categorized based on disease location and severity by the Paris pediatric modification of the Montreal classification of IBD (9). It is well-recognized that, in children, UC can present with atypical features, including macroscopic rectal sparing (5–30%), backwash ileitis in association with severe pancolitis, and limited distal disease associated with mild cecal inflammation with an otherwise normal right colon (cecal patch) (6, 9, 10). The presentation and natural history of pediatric UC are distinct from those of adult UC in that the majority of pediatric-onset UC presents with extensive colitis affecting the entire colon (10–13). On the contrary adults predominately present with left-sided colitis (14), with more than half in remission or with mild disease activity after initial presentation (14–16).

## Rationale for Precision Medicine in UC

Pediatric UC patients exhibit a wide variety of disease activity and heterogeneity in disease course. While pediatric UC patients often present with extensive colitis at first presentation, response to initial therapies, and disease course varies (17). Thus, it can be challenging to predict which patients will have progression of disease and require colectomy based on phenotypic description at presentation alone. Predictors that identify which treatments will be effective for which patients are critically needed so that patients can achieve rapid and sustained improvement in symptoms and quality of life (18), normalize growth and development (6), reduce cumulative exposure to corticosteroids and their associated toxicities, and avoid disease complications including toxic megacolon and colitis-associated colorectal cancer later in life (19, 20). Moreover, we wish to consistently use the safest treatment approach that will achieve this goal for any individual patient.

Recent studies suggest positioning more effective biologic therapies earlier in the disease course for patients at high risk for refractory disease to provide a more effective response, prior to establishing some form of chronicity (21–23). Although in Crohn's disease (CD), a distinct “window of opportunity” has been identified to initiate effective treatment prior to the development of irreversible bowel damage, there is much debate about whether this concept equally applies to UC (24). Although UC was historically thought to be a more superficial reversible disease, primarily impacting the mucosa and submucosa, more recent evidence suggests that the inflammatory burden in UC may also cause progressive bowel damage (23). Cumulative colonic damage may lead to remodeling and colonic fibrosis (22, 23), further supporting the notion that a step-up approach to treatment may not be optimal. We therefore need additional predictors of disease progression to guide optimal treatment strategies that are personalized based on patient and disease-related factors that can predict optimal therapy and prevent persistent inflammation and adverse disease outcomes.

## Optimal Clinical Endpoints

To evaluate the best predictors for each patient, we need to first establish the optimal clinical outcome measures and endpoints. Currently, in clinical practice, the most accepted endpoint is sustained and durable clinical corticosteroid-free remission, with a normal health-related quality of life and prevention of hospitalization, surgery, and cancer (25). While current pediatric guidelines strive for clinical remission, defined by a pediatric UC index (PUCAI) <10 (26), the most recent adult clinical guideline recognizes mucosal healing as a treatment goal (25). However, the pediatric guidelines recognize the importance of mucosal healing and indicate that the demonstration of mucosal healing is warranted prior to de-escalation of treatment (26). Most recently, adult data suggest that histological remission may be an important endpoint to consider (27). In 2015, the Selecting Therapeutic Targets in Inflammatory Bowel Disease (STRIDE) program developed goals for a “treat to target” approach in patients with IBD. These consensus-based recommendations advocated patient-reported outcome (PRO) evidence of remission and endoscopic healing as a treatment target in UC. Biomarker remission [normal C-reactive protein (CRP) and calprotectin] was considered an adjunctive target (28). In pediatric practice, although subsequent mucosal endoscopic evaluation is the gold standard to evaluate disease activity in response to therapy and is gaining acceptance as standard of care (29), current published pediatric consensus recommendations focus on clinical activity and biochemical evaluation with fecal calprotectin if in remission (26, 30).

## Predicting and Monitoring Response to Medical Therapy

Traditionally, the treatment choice for pediatric UC is determined based on disease activity classified as mild, moderate, or severe (30). However, full implementation of personalized medicine in pediatric UC requires (A) predictors or response to treatments with distinct mechanisms of actions to direct the right treatment to the right patient and (B) predictors of drug pharmacokinetics (PK) (such as what affects exposure and drug clearance) as well as PK and pharmacodynamic (PD) targets to optimize a specific therapy and ensure its sustained effect. Factors associated with response to medical or surgical therapy, discussed in more detail below, are listed in Table 1.

**Table 1:** Factors associated with response to therapy in pediatric UC.

**Therapy**	**Outcome**	**Predictors**
5-ASA ± corticosteroid induction therapy	Week 4 clinical remission	Total Mayo score < 10 (31)
		Proctosigmoiditis (31)
		Relative rectal sparing (31)
		Rectal biopsy eosinophils > 32/HPF (31)
		Higher serum albumin (31)
		Corticosteroid response gene signature (32)
	Week 12 corticosteroid-free clinical remission	PUCAI < 35 (31)
		Higher serum albumin (17)
		PUCAI < 10 at week 4 (17)
	Week 52 corticosteroid-free clinical remission	Baseline PUCAI < 45 (17)
		Baseline Hgb ≥ 10 g/dl (w/o week 4 remission) (17)
		PUCAI < 10 at week 4 (17)
		PUCAI < 10 at week 12 (17)
		Antimicrobial peptide gene signature (negative association) (17)
		Increased Clostridiales taxa (17)
	Escalation to anti-TNF therapy	Total Mayo score ≥ 11 (17)
		Rectal biopsy eosinophils ≤ 32/HPF (17)
		Low serum 25-OH vitamin D (17)
		Hgb < 10 g/dl (17)
		PUCAI ≥ 10 at week 4 (17)
		Cellular transport (inverse association) and antimicrobial gene signatures (17)
		Decreased Clostridiales taxa (17)
		Baseline PUCAI (17)
	Colectomy	Clostridiales, *Veillonella dispar, Haemophilus parainfluenzae*, and *Campylobacter* (33)
Initial therapy (heterogeneous)	Weeks 26 and 52 corticosteroid-free remission	Rectal type 2 immune response gene expression pattern (34)
Intravenous corticosteroid induction therapy	Week 12 corticosteroid-free remission on 5-ASA	Absence of surface villiform histologic changes (31)
	Treatment escalation or colectomy prior to discharge	Day 3 PUCAI > 45 (35)
		Day 5 PUCAI > 70 (35)
	Treatment escalation or colectomy by week 12	Surface villiform histologic changes (31)
		Rectal biopsy eosinophils ≤ 32/HPF (31)
	Day 5 PUCAI	Stool microbial Shannon diversity (36)
Colectomy and IPAA	Chronic pouchitis	PUCAI score (37)
		Cumulative corticosteroid (38)
		Peripheral blood neutrophils (%) (38)
		Surgeon performs < 10 IPAAs/year (39)

*5-ASA, 5-aminosalicylate; PUCAI, pediatric ulcerative colitis activity index; ASUC, acute severe ulcerative colitis; IPAA, ileoanal pouch anastomosis*.

### Response to Corticosteroids and 5-Aminosalicylic Acid (5-ASA)

As for any chronic condition, the optimal management for pediatric UC is to use the lowest dose of the safest therapy that is effective for maintaining full remission. For a child with UC, sustained corticosteroid-free remission on maintenance therapy with 5-ASAs is the ideal outcome. Prior studies have demonstrated that corticosteroid-free clinical remission with this strategy ranges between 38 and 40% (17, 40).

Historically, 5-ASA therapy with or without corticosteroids during induction of remission was the most common first-line strategy for the majority of mildly to moderately active UC prior to resorting to alternative “salvage therapy.” Predictors of responses to a combination of corticosteroids and 5-ASA regimens were evaluated in a multicenter retrospective cohort study (41). Interestingly, corticosteroid-free clinical remission 3 months after diagnosis, as evidenced by a PUCAI <10, was the strongest predictor for 1-year sustained corticosteroid-free clinical remission on 5-ASA. No baseline variables, including PUCAI, endoscopic evaluation, or laboratory serum markers, predicted corticosteroid-free remission or colectomy. However, baseline PUCAI did predict subsequent acute severe colitis and need for salvage therapy.

Response to 5-ASA with or without corticosteroid induction therapy was studied prospectively in the landmark multicenter inception cohort study, the Predictors of Response to Standardized Pediatric Colitis Therapy (PROTECT) study (17, 34). In this study, treatment at diagnosis was stratified based on mild (mesalamine alone), moderate to severe (oral corticosteroid induction plus mesalamine maintenance therapy), or severe to fulminant (hospitalization and IV corticosteroid induction plus mesalamine maintenance therapy) clinical disease severity. Predictors of corticosteroid-free clinical remission (PUCAI < 10) without treatment escalation or colectomy and need for treatment escalation to anti-tumor necrosis factor (TNF) therapy were assessed at 12 weeks and 1 year (17, 31). After 1 year of follow-up, 38% of patients were in corticosteroid-free clinical remission without further treatment escalation and remained treated with mesalamine (98%) or no therapy (2%) (17). Clinical predictors of corticosteroid-free remission at 1 year included a baseline PUCAI < 45, hemoglobin of at least 10 g/dl (without week 4 remission), and week 4 remission (Tables 1, 2). The range of likelihoods of achieving corticosteroid-free remission without treatment escalation based on representative patient factors using the PROTECT model is shown in Table 2. Translated into clinical practice, this means that early reassessment of disease as soon as 4 weeks after mesalamine with or without corticosteroid induction is critically important. Treatment escalation should be strongly considered if there is no clinical remission at that time and if, at baseline, hemoglobin was <10 g/dl or PUCAI was ≥45 given the low probability of corticosteroid-free remission at 1 year in patients with these characteristics (Table 2).

**Table 2:** Predicted probability of week 52 outcomes for select patient scenarios based on the PROTECT study predictive models.

**Outcome**	**PUCAI**	**Hgb (g/dl)**	**Week 4 remission**	**Mayo score**	**Rectal Eos (/HPF)**	**25-OH-D (ng/ml)**	**Probability**
**CSFR**							
Most likely	<45	–	Yes	–	–	–	0.60
Least likely	≥45	<10	No	–	–	–	0.08
Escalation to Anti-TNF^*^							
Most likely	–	<10	No	≥11	≤32	<20	0.96
Least likely	–	≥10	Yes	<11	>32	≥30	0.13

* *In patients with moderate to severe disease activity at diagnosis*.

*CSFR, corticosteroid-free remission with escalation of therapy beyond mesalamine; Hgb, hemoglobin; Eos, eosinophils; 25-OH-D, 25-hydroxy vitamin D. Probabilities calculated from the PROTECT study regression formulas (17)*.

### Escalation to Anti-TNF Therapy

The most useful clinical predictors of escalation to anti-TNF therapy were identified in the PROTECT study (17, 31). In PROTECT, among patients with moderate to severe disease, a total Mayo score of at least 11 was predictive of escalation to anti-TNF therapy by week 52, while rectal eosinophils of at least 32 per high-power field (HPF), higher 25-OH-vitamin D levels (≥30 ng/ml better than 20 to <30, which was better than <20), hemoglobin of at least 10 g/dl, and week 4 remission were clinical predictors protective of anti-TNF treatment escalation. A patient with a total Mayo score of 11 or greater with no protective factors had a predicted probability of escalation to anti-TNF therapy of 0.96. On the contrary, a patient with a total Mayo score of <11 and all other protective factors had a predicted probability to escalate to anti-TNF therapy of 0.20 (Table 2). Therefore, in addition to the previously identified baseline factors, patients who have severe endoscopic disease and do not achieve remission by week 4 should also be considered for early escalation to anti-TNF therapy. At 1 year, about half (48%) of all patients who escalated to anti-TNF therapy were in corticosteroid-free clinical remission. PROTECT also illustrated that a large proportion of baseline moderate to severe patients (43%) escalated early to anti-TNF within the first 4 weeks, while no baseline mild patients escalated to anti-TNF within the 1st month. However, the rate of escalation to anti-TNF after the first 4 weeks was similar between the mild and moderate to severe groups. These clinical predictors provide a good framework to anticipate the need for escalation, and decisions regarding the optimal timing of escalation should take into account the window of opportunity to maximize the benefit of anti-TNFs.

### Response to Anti-TNF Therapy

When assessing for predictors of response of anti-TNFs, it is important to evaluate the overall efficacy of this class of therapy. A randomized open-label parallel group multicenter study (the T72 trial) for moderate to severe pediatric UC patients that compared 5 mg/kg infliximab (IFX) every 8 weeks vs. every 12 weeks of dosing found that at week 8, 73.3% of patients who were previously refractory to conventional (nonbiologic) therapy had a clinical response by Mayo score (42). At 1 year of therapy, 28.6% of patients were in clinical remission as assessed by the PUCAI. A *post hoc* analysis identified that week 8 PUCAI scores were the strongest predictors of 1-year corticosteroid-free remission (43). Moreover, clinical remission defined by a PUCAI score <10 had a high correlation with mucosal healing at week 8; however, mucosal healing at week 8 did not predict 1-year corticosteroid-free remission in this analysis. One should keep in mind that these predictors were identified in an early clinical trial phase, and response and predictors of response may change while practicing either concomitant combination therapy, as well as with the implementation of proactive therapeutic drug monitoring (44). Furthermore, to our knowledge, no pediatric studies have identified predictors of mucosal healing or deeper histologic healing after IFX therapy.

While these initial studies evaluated the efficacy and predictors of labeled 5 mg/kg dosing, it is well-established that some patients benefit from intensified dosing strategies. An international survey conducted with pediatric gastroenterologists showed that escalated dosing up to 10 mg/kg with shorter than labeled dosing intervals for pediatric UC was common practice (45). While respondents identified that dose escalation was mostly indicated for acute severe UC (ASUC), which is discussed in detail below, between a third to half of the respondents found this indicated in moderate to severe disease.

There is further evidence that precision anti-TNF dosing based on individual patient factors and PK is likely beneficial in pediatric UC. Our group has demonstrated, in two retrospective pediatric IBD studies, that anti-TNF dosing based on PK models created from these covariates may be more beneficial than standard labeled dosing and that proactive anti-TNF drug monitoring can improve outcomes when applied across a large practice (44, 46). In fact, PK analysis from the T72 trial identified that among children with moderately to severely active UC, higher quartile post-induction IFX levels ≥41.1 μg/ml at week 8 as a marker of drug exposure were associated with a higher numerical difference of clinical response, mucosal healing, and clinical remission (based on a Mayo score), compared to patients with a lower level (47). Importantly, however, this study did not report if these changes were statistically significant (47). A more recent retrospective study confirmed that higher post-induction IFX trough level quartiles were associated with clinical, biological, and combined remission (48). Another important PK property, immunogenicity, might be predicted by genetic variants. A genome-wide association study (GWAS) of the combined pediatric and adult British PANTS cohort identified an association between HLA-DQA1^*^105 and anti-TNFs as well as immunogenicity for CD, and this finding remained true in a subanalysis of a replication cohort with UC patients only (49). In summary, these data suggest that precision dosing of IFX in clinical practice should take into account these individual PK predictors that are associated with increased drug clearance [such as albumin <3.5 g/dl, erythrocyte sedimentation rate (ESR) >9 mm/h, and antibodies to IFX (ATIs)] in addition to weight, and subsequent dosing decisions should be based on proactive monitoring of therapeutic drug levels (44, 46).

The second most commonly used anti-TNF therapy, the subcutaneously administered humanized monoclonal antibody adalimumab, is currently labeled by the Food and Drug Administration (FDA) for adult but not pediatric UC. While adalimumab is used off-label in the pediatric setting, there is limited data for pediatric UC (50). A retrospective study that included patients who had previously failed IFX found that 41% of patients were in corticosteroid-free remission after 1 year of therapy and that 28% had mucosal healing (51). This study did not find any significant differences between IFX nonresponders and patients intolerant to IFX.

### Response to Other Biologic and Small-Molecule Drugs

Vedolizumab (VDZ), an α4β7 anti-integrin inhibitor, is from a second biologic class that was approved for the use of adult IBD in 2014. The first pediatric retrospective study of VDZ identified that 76% of UC patients were in remission at week 14. In a subanalysis at week 22 that included both patients with UC and CD, anti-TNF-naïve patients were more likely to be in remission than anti-TNF-exposed patients (52). Another retrospective pediatric study demonstrated that 59% of anti-TNF-naïve patients with UC achieved endoscopic remission, compared to 15% of anti-TNF-exposed patients. However, the authors noted that anti-TNF-naïve patients had lower partial Mayo baseline scores compared with exposed patients (53). The anti-TNF exposure findings were similar to the original adult GEMINI study in which anti-TNF-naïve patients had faster symptom improvement (54), and a subsequent network meta-analysis confirmed that more anti-TNF-naïve patients achieved remission compared with anti-TNF-exposed patients (55).

Only sparse data are available regarding PK predictors of response to VDZ in pediatric UC. A retrospective analysis of pediatric VDZ trough levels identified that a lower baseline albumin, a higher fecal calprotectin while on treatment, an elevated CRP, and longer time between infusions were associated with lower levels among pediatric IBD patients (56). While this analysis did not stratify between UC and CD patients, UC patients had higher trough levels compared with CD patients. While this paper did not find trough levels to be predictors of response, the definition of response was sustained use of VDZ as a proxy of maintenance of remission. The prospective VEDOKIDS study (NCT02862132) is currently being conducted to evaluate further predictors of response (57).

Even fewer pediatric-specific predictors of response are known for anti-TNF therapy beyond IFX and adalimumab such as golimumab (58) or other biologic classes not yet approved for pediatric UC beyond anti-TNF and VDZ or biosimilars or novel small molecules such as Janus kinase inhibitors.

## Specific UC Presentations and Phenotypes

### Very-Early-Onset UC

The youngest patients with UC form a unique subpopulation who potentially most warrant a personalized approach. The Paris classification defines early-onset IBD (EO-IBD) as those children diagnosed prior to age 10. Those diagnosed under the age of 6 years have been further defined as very-early-onset IBD (VEO-IBD), and those diagnosed under 2 years of age are characterized as infantile IBD. The incidence of IBD in children under 5 is rising with an annual percentage increase of around 7% (59, 60). Children with VEO-IBD, regardless of a CD or UC diagnosis, more often have colonic localization of the disease (12). Accordingly, a majority of patients with VEO-IBD are diagnosed with either UC or IBD-U (12, 61, 62). Advances in next-generation sequencing technologies have led to earlier detection of monogenic causes of VEO-IBD. Still, monogenic etiologies are only identified in ~8% of all VEO-IBD cases. Importantly though, children diagnosed under the age of 2 (infantile IBD) have 6 times the odds of having a monogenic disease (63). Genetic mutations that have been identified in VEO-IBD patients with a UC-like presentation include those in genes related to epithelial function (EPCAM, GUCY2C, and TTC7A), immune cell activation (LRBA and STAT1), immune cell regulation (IL10RB), and hyperinflammatory disorders (XIAP and HPS1) (63, 64). In patients with UC-like monogenic IBD, there are often extraintestinal abnormalities that can be identified on history, physician exam, or basic laboratory workup such as a history of recurrent infections, skin disorders, or leukopenia. Several options are clinically available for broad genetic testing, including whole-exome or whole-genome sequencing and targeted gene sequencing panels. Making a diagnosis of a monogenic cause of UC is critical as stem cell transplant can be curative for some deficiencies (IL10 receptor and XIAP) or the deficiency may be directly targeted by medical therapy (65, 66), such as with abatacept for LRBA deficiency (67). Therefore, comprehensive testing for immune function and phenotype in parallel with genetic testing should be performed in children with a VEO-IBD under the age of 2 and should be considered in children under 5 or older, particularly those with other autoimmune disorders or immune abnormalities (68–70).

While the overall natural history of most VEO-UC does not appear to be more severe than later-onset UC, some important observations have been made with regard to treatment. Population-based and multicenter retrospective studies reveal that disease severity and colectomy rates are similar in patients with UC-like VEO-IBD compared to older pediatric UC patients. More recent data suggest that the risk of colectomy among VEO-IBD is similar (14%) to that in patients diagnosed with UC at an older age (12, 59, 62). While patients with VEO-UC do not appear to have a higher likelihood of escalating to IFX therapy (12, 62), they may have unique characteristics influencing the durability of the effect of IFX. IFX dosing is weight based, but weight exhibits a nonlinear relationship with IFX clearance such that smaller patients treated with the same weight-based dosing are more likely to be exposed to less drug with lower trough levels (71). Therefore, it should not be surprising that studies examining response to IFX treatment in EO-IBD (including those with UC and IBD-U) have observed a higher likelihood of anti-TNF failure, need for anti-TNF dose intensification, and ATI formation (72, 73). Proactive therapeutic drug monitoring is particularly important in these young patients as conventional dosing likely results to suboptimal exposure leading to higher risk of immunogenicity and loss of response.

### Acute Severe UC

ASUC is a life-threatening condition originally defined by Truelove and Witts as a severe exacerbation of UC with at least six daily bloody bowel movements and at least one of several signs or laboratory indicators of hemodynamic instability or severe systemic inflammation (74). In pediatrics, a contemporary definition of ASUC is UC with sufficiently severe diarrhea, hematochezia, abdominal pain, and activity limitation to indicate a PUCAI score of at least 65 points (75). Approximately 25% of children with UC will require hospitalization for an ASUC exacerbation (76). Patients are generally first treated with intravenous corticosteroids, resulting in a response in 63–74% of patients (31, 35). In the age of biologic therapy, induction treatment with intravenous, followed by oral, corticosteroids remains appropriate, because 21% of pediatric patients presenting with ASUC treated with intravenous corticosteroids will ultimately achieve corticosteroid-free clinical remission on 5-ASA drugs alone (31).

Baseline factors alone remain overall poorly predictive of response to corticosteroid therapy in ASUC, but early clinical assessments with PUCAI are a mainstay for predicting the need for rescue therapy. In the subgroup of patients in the PROTECT UC inception cohort with disease sufficiently severe to warrant intravenous corticosteroid treatment, among all baseline clinical, laboratory, and histopathologic features, only absence of surface villiform change on histology (villous-like appearance of the colon surface epithelium) was associated with week 12 corticosteroid-free remission on 5-ASA (31). Both surface villiform changes and low rectal biopsy eosinophils were associated with additional therapy or colectomy by week 12. Although baseline clinical and laboratory parameters are not associated with clinical response to corticosteroids in pediatric ASUC, early monitoring of symptoms with the simple weighted PUCAI score has been prospectively validated as a predictor of response to corticosteroids and incorporated into society guidelines (26). Patients with a PUCAI score of 45 or less on day 3 of corticosteroid treatment have a 94% chance of successful discharge without rescue therapy, whereas by day 5, patients with a PUCAI score >65 are 82% likely to require rescue therapy (35). Levels of stool inflammatory markers, osteoprotegerin and M2 pyruvate kinase, and serum IL6 were shown to be predictive at day 3 of the need for rescue therapy in ASUC, but none were superior to the PUCAI clinical score (77–79). Integration of PUCAI scores into the daily clinical monitoring of children admitted with ASUC should limit the time to determine the need for rescue therapy with IFX to no more than 5–7 days.

A pilot randomized controlled study demonstrated that intravenous corticosteroids plus an oral antibiotic cocktail resulted in a larger clinical response by day 5 (as measured by day 5 PUCAI) compared to corticosteroids alone in pediatric patients with ASUC (36). The study was not powered to detect differences in the incidence of rescue therapy or colectomy. At baseline, microbial diversity was severely restricted. While high microbial diversity was associated with higher day 5 PUCAI in the intravenous corticosteroid group, no such association was observed in the intravenous corticosteroid plus antibiotic group. In this relatively small trial, there were also no associations detected between bacterial taxa and response to intravenous corticosteroid plus antibiotics. Although the addition of oral antibiotics to intravenous corticosteroids may result in improved early clinical responses in pediatric ASUC, microbial predictors of which patients are most likely to benefit remain elusive.

The anti-TNF biologic drug IFX is the mainstay of rescue therapy for ASUC in pediatric patients whose disease is unresponsive to intravenous corticosteroids. Approximately 75% of children with ASUC rescued with IFX will initially respond. By 1 year, 23–45% will require a colectomy (76, 80). As with corticosteroid therapy, no baseline clinical or laboratory factors have been strongly associated with response to anti-TNF therapy in ASUC. Patients with ASUC exhibit more rapid clearance of IFX, likely related to high TNF burden, upregulation of reticuloendothelial system clearance by the immune system, and loss of the drug through the leaky inflamed bowel (81). In adults with ASUC, more rapid IFX clearance is associated with treatment nonresponse (82, 83). Consistent with likely having high drug clearance, children hospitalized with severe colitis have a high incidence of infusion dose or frequency escalation. Baseline high ESR, a marker of systemic inflammation, and low albumin, a marker of gut protein loss, are associated with the eventual need for dose escalation in this population (84). A recent retrospective comparative effectiveness study found that intensified IFX induction dosing in children hospitalized with corticosteroid-refractory UC was associated with improved rates of clinical remission and colectomy (80). It follows that children with ASUC, especially those with hypoalbuminemia, should be treated with intensified dosing and with proactive therapeutic drug level monitoring.

### PSC-IBD Phenotype

Primary sclerosing cholangitis (PSC) is an uncommon disease, and even rarer in children, with an incidence of 0.2 per 10^5^ person year (PY) and a prevalence of 1.5 per 10^5^ PY (85, 86), and typically presents in the second decade of life. Analogous to the adult literature, the majority (~75%) of children with PSC will develop concomitant IBD, mostly UC or IBD unclassified (IBD-U) (83%) (87, 88). By contrast, it remains uncommon for both adults and children with IBD to develop concomitant PSC (1.6–6.8%) (85, 89). There is increasing evidence in pediatrics that IBD coexisting with PSC (PSC-IBD) has a distinct IBD phenotype. Typically, PSC-IBD is characterized by pancolitis with a predilection for worse disease in the right colon, relative rectal sparing, and less commonly backwash ileitis (88–91). Another key distinguishing feature of PSC-IBD phenotype is the increased risk of colorectal carcinoma (CRC). A large meta-analysis found that PSC-IBD patients had a threefold increase of CRC compared to IBD-only patients (92). The current pediatric UC ECCO/ESPGHAN guidelines advise surveillance colonoscopy every 1–2 years after diagnosis in adolescents (>12 years of age) with PSC-IBD (30). Paradoxically, PSC-IBD patients have a milder clinical disease course (93). However, it is becoming apparent that clinical symptoms in PSC-IBD underrepresent mucosal inflammation (93). Ricciuto et al. (94) showed in a prospective study that children with PSC-IBD in clinical remission (PUCAI < 10) had a significantly higher risk of active endoscopic disease than children with UC without PSC. Certainly, the observed discordance in symptoms and mucosal disease warrants maintaining a lower threshold for objective monitoring with fecal calprotectin or endoscopic evaluation patients with PSC-IBD. It is noteworthy that pediatric PSC has a chronic progressive course in children, with over 50% of children in the PSC consortium developing adverse liver outcomes after 10 years (portal hypertension, biliary complication, and liver transplantation) (87). Given the postulated bidirectional interplay of the gut–liver axis, it has been proposed that elimination of colonic inflammation by optimizing IBD care or colectomy may be associated with a milder PSC course; however, findings in adult cohorts to date have been conflicting (95, 96). The underlying pathophysiology of PSC-IBD remains to be elucidated, but one hypothesis is that gut-homing lymphocytes aberrantly traffic to the liver (97). This had led to interest in drugs that interfere with T-lymphocyte trafficking such as VDZ. Most recently, a large adult PSC and IBD international study group found no evidence of liver biochemical response to VDZ, and the overall IBD response was comparable (98). Likewise, in 37 pediatric PSC-IBD patients, VDZ did not improve liver biochemistry (99).

### Complications After Colectomy and Ileal Pouch Anal Anastomosis (IPAA)

Within 5 years of diagnosis, 12–15% of children with UC will have disease refractory to medical therapy and require total colectomy and IPAA (3, 100, 101). In this staged surgical procedure, the diseased colon and rectum are removed, and a pouch reservoir is constructed from the distal ileum and anastomosed to a short rectal cuff to preserve continuity and avoid a permanent ileostomy. Although outcomes after colectomy and IPAA in pediatric patients are generally quite good, inflammatory complications such as acute pouchitis (30–60%), chronic pouchitis (15–35%), and *de novo* CD of the pouch (13–25%) can occur (37, 38, 102–107). Rates of overall pouch failure are fortunately quite low at ~8% (105).

Attempts at identifying predictors of chronic pouchitis or *de novo* CD in children with IPAA have generally been limited by mostly retrospective single-center designs with relatively small sample sizes. Several factors either directly related or likely related to preoperative disease severity including PUCAI score, preoperative cumulative corticosteroid use, and preoperative peripheral blood neutrophil percentage have been associated with higher risk for development of chronic pouchitis in children (38, 108).

One multicenter retrospective cohort identified a distinctly modifiable risk factor, surgeon experience, with risk of chronic pouchitis. Children who underwent colectomy and IPAA by a surgeon who performs fewer than 10 procedures per year had a significantly increased likelihood of developing chronic pouchitis over time (39). Equally frustrating is that no risk factors have been identified that predict the development of *de novo* CD of the pouch in children. Children diagnosed with CD under 6 years of age are more likely to have a UC-like presentation with disease only involving the colon (61, 62), placing pediatric gastroenterologists theoretically at risk of misdiagnosing young children with CD as UC. For this reason, more children in this age group are diagnosed as IBD-U. However, two single-center cohort studies did not detect an increased risk of *de novo* CD of the pouch with younger age of UC diagnosis (103, 107). A limitation of these studies is the small number of patients diagnosed at younger than 6 years of age. Larger multicenter cohorts will be needed to determine if young age at UC diagnosis is a risk factor for *de novo* CD of the pouch. It remains prudent to delay IPAA in young children with IBD undergoing colectomy, especially if a diagnosis of UC is at all uncertain.

## Molecular and Microbial Predictors of Response

The rapid pace of technologic innovation over the past few decades allows us to characterize patient cellular, molecular, and microbial makeup at an unprecedented scale. A major research goal is to identify and integrate patient biologic and microbial signatures to accurately predict treatment response. The hope is that, in the future, rather than using the current trial-and-error approach to treatment, physicians could send off a rectal biopsy, stool, or blood sample for analysis that predicts the likelihood that a patient will respond to 5-ASA, anti-TNF, anti-integrin, or other emerging biologic or small-molecule therapies. Excitingly, recent large translational pediatric cohort studies are taking us a step closer to making this futuristic goal a reality (17, 31, 34).

Early immunologic studies of adult IBD identified a type 2 immune response, typical of allergy or response to parasites, specific to UC and not CD (109). Our group studied the mucosal immune response in treatment-naïve pediatric UC and CD patients from the Risk Stratification and Identification of Immunogenetic and Microbial Markers of Rapid Disease Progression in Children with Crohn's Disease (RISK) Study (110). We determined that, at diagnosis, pediatric UC patients can be distinguished from those with Crohn's colitis based on the expression of genes related to type 2 (IL13 and IL5) and type 17 (IL23) immune responses (34). Interestingly, a type 2 gene expression pattern was associated with a higher likelihood of corticosteroid-free clinical remission at 6 and 12 months after diagnosis in pediatric UC. Most of the patients in this study were treated with corticosteroids, 5-ASA, and thiopurines, with a very few exposed to IFX. This finding will need to be validated in a larger cohort with better-defined treatment exposures but suggests that a more robust type 2 mucosal immune response may be associated with response to initial drug treatment such as corticosteroids or 5-ASA. Eosinophils are a cell type associated with allergy and type 2 inflammation. Our observation of an association of type 2 inflammation with improved treatment response is in line with findings from the PROTECT study that higher mucosal eosinophil count is associated with a lower likelihood of escalation to anti-TNF therapy (17, 31).

While our aforementioned study examined a panel of targeted genes, others have cast a much wider net with genome-wide approaches. A North American investigative group compared peripheral blood global gene expression at day 3 of intravenous corticosteroid treatment between extreme responders and nonresponders using an RNA microarray. They identified a 41-gene panel with 80% sensitivity and specificity for corticosteroid response (111). The PROTECT study investigators applied tissue RNA sequencing and 16S ribosomal RNA sequencing to describe the entirety of host genes expressed and the microbial composition and identify gene expression and microbial predictors of response to therapy in UC. Early remission at week 4 was the strongest clinical predictor of later treatment response, so they initially analyzed gene expression predictors of week 4 response to corticosteroids. They identified a signature composed of 115 genes that differentiated early responders from nonresponders and augmented a predictive model based on clinical and histopathologic features alone (31). Corticosteroid response genes were involved in the innate immune response (neutrophils and macrophages), response to bacteria, and immune cell recruitment (32).

In an evaluation of later outcomes, PROTECT investigators identified a 33-gene signature that associated with corticosteroid-free clinical remission without escalation to 5-ASA at 52 weeks (17). This signature was composed of 18 genes related to the transport of electrolytes and nutrients at the epithelium with increased expression in remitters and 15 genes related to antimicrobial responses (antimicrobial peptides) that were decreased in remitters. When incorporated into multivariable models, the transport signature best predicted week 52 corticosteroid-free remission, while both signatures predicted escalation to anti-TNF therapy in patients with moderate to severe UC. Microbiome analyses identified increased abundance of bacterial taxa from the Clostridiales order, generally considered to be associated with gut health, in patients who achieved corticosteroid-free remission at 52 weeks and decreased abundance in those who escalated to anti-TNF therapy. The abundance of three taxa of oral microbes was associated with progression to colectomy, and the reduction of these taxa with treatment was associated with corticosteroid-free remission at 52 weeks (33). The integration of the abundance of specific Clostridiales taxa and gene expression signatures improved clinical prediction models for both 52-week corticosteroid-free remission and escalation to anti-TNF therapy in the PROTECT study.

## Future Applications of Machine Learning

Machine learning has the potential to change practice in UC, a chronic disease as described above that has a variable disease course in individuals. Advances have been made in next-generation sequencing, and high-throughput “omics,” leading to a greater understanding of the molecular basis of pediatric UC. Innovations in the application of machine learning to derive not only automated learning of relevant features but also learning from patterns that are not obvious to human vision will advance the field further. Our reliance on multiple modalities such as endoscopy, histology, and imaging to ascertain the diagnosis and to monitor disease progression positions our field to capitalize on advances in machine learning. In leveraging computational approaches that can analyze large multimodal data, we could truly translate established and newly discovered predictive factors into the clinical setting. Incorporating a clinical decision support tool that, for example, supports patient stratification at disease onset and allocation of personalized therapies, all within the electronic health record, could provide data-driven solutions for individual patient encounters.

In the literature, the terms artificial intelligence (AI) and machine learning are often used interchangeably, despite notable differences. AI is a broad concept referring to computer programs that are able to mimic human cognitive function, such as the ability to “learn” (112). Machine learning is an application of AI and refers to the ability of a computer to learn from the data without explicit programming. It refers to a system that can develop an algorithm to make a prediction by recognizing patterns (feature) relating to that data for a specific prediction (112), which can be achieved by either supervised or unsupervised learning. A great deal of research to date in gastroenterology has focused on computer vision. Computer vision refers to computer systems processing images or videos so that some form of understanding from the content can be acquired (e.g., endoscopy, video capsule endoscopy, and radiologic imaging) (113).

Advances in computational methods will mostly likely lead to automated image analysis and recognition to enhance expert-level interpretations of endoscopic, histologic, or cross-sectional images (114). Proof-of-concept studies in adults have supported the potential use of deep learning (convolutional neural networks) in replicating endoscopic disease severity in UC by delineating remission from moderate and severe disease, with high accuracy (115). Also, recent models based on endoscopic still images were found to predict histologic remission with 92.9% accuracy in UC (114). It is yet to be determined whether such approaches will for example reliably predict disease severity using endoscopic videos, with models to date built using still images.

Machine learning holds much promise, but prior to implementation in the clinical setting, there are considerable challenges to overcome. Models are intrinsically dependent on the training dataset and will incorporate any bias, subjectivity, and variation contained in that training dataset; thus, models may not be generalizable to the wider population. Moreover, findings often lack explainability (“black-box”), particularly in models derived from deep neural network algorithms (a type of machine learning algorithm that employs complex artificial neural networks that automatically detect and transform relevant features into learning). Great care is required in standardizing, selecting the training set, and qualifying the reference standard with a gold standard. AI in the future will be integral, and the keys to successful implementation of this technology in clinical care will be engaging key stakeholders, regulatory and professional society, and practicing physicians to develop governance and ethical standard.

## Case-Based Conceptualization of Pediatric UC Precision Medicine

**Figure 1:**
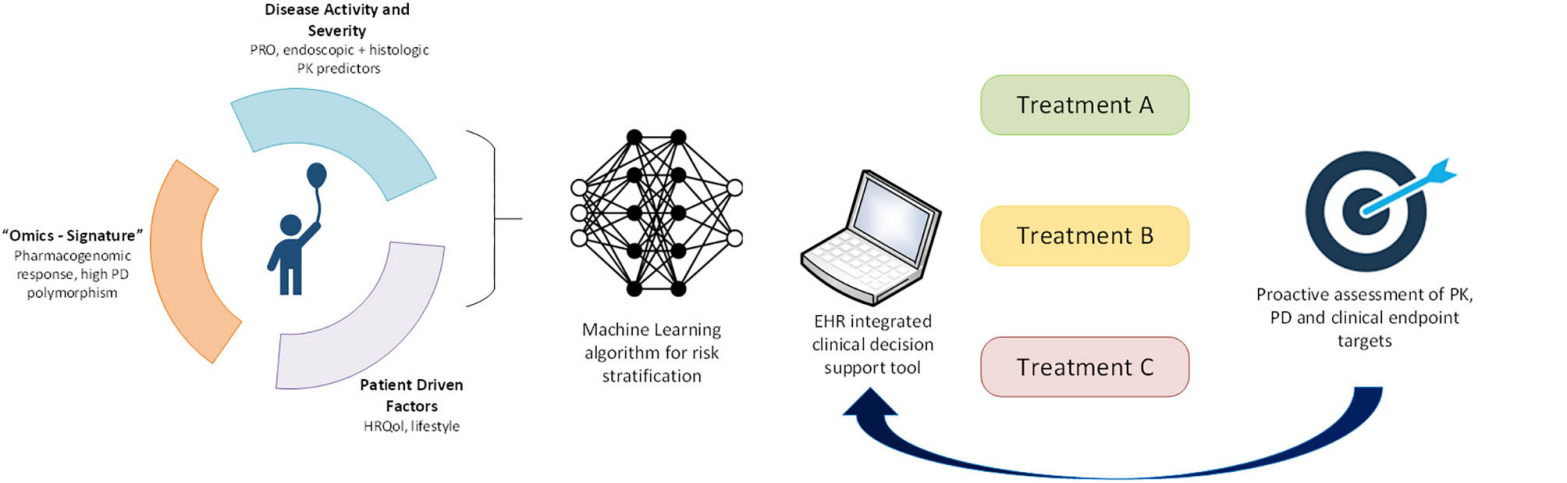
Application of precision medicine to individualize pediatric UC care. In the future, we will leverage machine learning approaches to derive a multimodal risk model to stratify individual patients to guide treatment choice. Longitudinal proactive integrated assessment of drug PK, PD biomarkers, and clinical endpoints in a clinical decision support tool within electronic health records will further support individualization of patient care.

In order to provide a practical demonstration of how precision medicine may be applied to pediatric UC now and in the future, we will apply concepts discussed in this article to a representative patient case. A 9-year-old girl is diagnosed with UC after ileocolonoscopy reveals diffuse moderate inflammation to the hepatic flexure (endoscopic Mayo score of 2). She has moderate clinical disease activity, with a PUCAI of 50 and a total Mayo score of 8. Laboratory evaluation at presentation reveals a hemoglobin of 9.5 g/dl and serum 25-OH vitamin D of 15 ng/ml. Histopathology reveals few rectal eosinophils (10/HPF). She is started on oral corticosteroids with a tapering dose and mesalamine. At a 4-week follow-up evaluation, she continues to have a clinically active disease with a PUCAI of 30. Based on models from the PROTECT study, her absence of remission at week 4, baseline PUCAI ≥ 45, and baseline hemoglobin < 10 g/dl predict she will be unlikely to achieve corticosteroid-free clinical remission on mesalamine. Similarly, her absence of remission at week 4, baseline 25-OH-vitamin D < 20 ng/ml, hemoglobin < 10 g/dl, and rectal eosinophil count < 32/HPF make her quite likely to require escalation to IFX (17). Therefore, our current knowledge suggests that initiation of IFX at this point would be timely and appropriate. In the future, these predictive clinical data points may be automatically extracted from the electronic medical record (EMR) and entered into models within an electronic clinical decision support (CDS) tool, embedded in the EMR, that provides best-practice alerts (Figure 1). We also envisage that microbiome, genomic, transcriptomic, and other “omics” data will be routinely generated as part of clinical care and integrated into the CDS tool to inform decisions, not just of when to start second-line therapy but also to predict which second-line therapy is most likely to be effective. In addition, machine learning will be applied to clinical, multiomic, and image (endoscopy, radiology, and histology) data to refine and update these predictions.

At the time of starting IFX, our patient's albumin remains low at 3.0 g/dl. Based on the established strong inverse relationship of albumin with IFX clearance, she is started at a dose of 10 mg/kg at 0, 2, and 6 weeks. In the future, a CDS tool may also enter current laboratory and anthropometric data into a PK dashboard to generate personalized IFX dosing regimens for each patient with high accuracy for achieving target induction and maintenance trough concentrations (116). She exhibits a strong clinical response to IFX with a PUCAI of 0 after 4 weeks of therapy. A trough IFX concentration at week 10, prior to the third induction dose, is 10 μg/ml. She is kept on a 4-weekly dosing to maintain this trough level. Twenty-six weeks after IFX initiation, she remains in clinical remission with a serum albumin of 3.8 g/dl, fecal calprotectin of 45 μg/g, and trough IFX level of 18 μg/ml at 4-weekly dosing with no detectible anti-IFX antibodies. The PK dashboard within the CDS tool predicts that spacing the dosing interval to every 6 weeks while maintaining the current dose will produce a trough level of 10 μg/ml; the dosing interval is changed accordingly. At 1 year, she remains in clinical remission and undergoes a flexible sigmoidoscopy demonstrating mucosal healing (endoscopic Mayo score of 0).

## Discussion

We stand at the threshold of an era in which we can implement personalized treatment strategies for our UC patients. In fact, well-designed pediatric UC cohort studies have provided us with information we can readily incorporate into clinical care. PUCAI has long been recognized as an important clinical tool in the management of ASUC in hospitalized children, and assessment of PUCAI at days 3 and 5 of intravenous corticosteroid therapy should prompt efficient identification of nonresponders in need of treatment escalation (35). For the broader group of all recently diagnosed children with UC patients, the PROTECT study has taught us that 1 month into treatment is a critical juncture. Those patients starting with a low hemoglobin or high PUCAI or Mayo score who do not achieve 1-month clinical remission have a low chance of attaining a corticosteroid-free remission at 1 year (17). It follows that prompt escalation to IFX therapy in these patients should be strongly considered. Once a patient is escalated to anti-TNF therapy, high disease severity and low albumin may be used to identify those at risk for rapid clearance and in need of intensified initial dosing. Rationally timed proactive assessment of anti-TNF PK, with drug levels, and PD, with fecal calprotectin, can inform precision dosing.

In addition to clinical predictors of treatment response, the PROTECT study has introduced histopathologic, molecular, and microbial predictors of response. For full realization of precision medicine in UC, multimodal patient data will need to be integrated through machine learning algorithms that interface with CDS tools embedded into the electronic health record to assign personalized therapy (Figure 1). Biomarkers of treatment PK (e.g., drug levels) and PD (e.g., fecal calprotectin or novel biomarkers) must provide feedback to these decision support tools to further individualize each treatment. Recent rapid advances in “omics” technologies, big data, and machine learning afford us this opportunity to shepherd the management of pediatric UC into the precision medicine era.

## Author Contributions

MR conceived of the manuscript. MR, RC, and JD drafted the manuscript and edited it for important intellectual content. All authors contributed to the article and approved the submitted version.

## Conflict of Interest

MR has served on an advisory board for Entasis Therapeutics. The remaining authors declare that the research was conducted in the absence of any commercial or financial relationships that could be construed as a potential conflict of interest.
